# Anaesthetic Techniques for Cardiac Ablation—A Scoping Review

**DOI:** 10.1111/aas.70323

**Published:** 2026-08-27

**Authors:** Mathilde Bang Fredensborg, Anne Færgemand Præstegaard, Sebastian Bach Fiege, Ann M. Møller

**Affiliations:** ^1^ Herlev Anaesthesia Critical and Emergency Care Science Unit (ACES), Department of Anaesthesiology Copenhagen University Hospital – Herlev and Gentofte Hospital Herlev Denmark; ^2^ Department of Clinical Medicine, Faculty of Health Sciences University of Copenhagen Copenhagen Denmark

## Abstract

**Background:**

Cardiac arrhythmias, particularly atrial fibrillation, have rising prevalence and represent a growing global health burden. Catheter ablation is a commonly used treatment demanding sedation or anaesthesia for comfort, stability and safety. Anaesthetic techniques vary globally, and the optimal strategy has yet to be determined. The objective of this scoping review was to map the current literature on anaesthesia for catheter ablation, identifying how it has been investigated and which evidence gaps exist.

**Methods:**

This scoping review followed a pre‐published protocol and guidelines. A search was conducted in MEDLINE, EMBASE, Cochrane, Google Scholar, and Scopus, with no restrictions on publication year or language. Eligible studies were primary studies involving adult patients undergoing catheter ablation in hospitals, clinics, or similar healthcare facilities.

**Results:**

We included 110 studies published between 1991 and 2025. The reported anaesthetic techniques included General Anaesthesia (GA), Deep Sedation (DS), Conscious Sedation (CS), Minimal Sedation, and Local Anaesthesia (LA). Complications were infrequently reported, recovery was comparable, and patient and operator satisfaction was high across techniques. Techniques varied across regions, with GA being most frequent in North America, GA and CS in Asia, and DS in Europe. Anaesthesia quality was commonly assessed by intraprocedural respiratory and haemodynamic stability and complication rates, whilst anaesthesia effectiveness was mostly assessed using procedure duration, accumulated medication, sedation depth, and sedation failure/escalation.

**Conclusion:**

This scoping review mapped current anaesthetic practices in catheter ablations. GA, DS, and CS were most frequently reported. Complications were infrequent and satisfaction high. However, evidence gaps and inconsistent definitions and assessment methods highlight the need for future research and standardisation.

**Editorial Comment:**

Electrophysiological cardiac interventions such as catheter ablations commonly now also involve anaesthesiological management. This scoping review presents an update on knowledge for how this care may facilitate good procedural results.

## 1 Introduction

Cardiovascular diseases remain the leading cause of morbidity and mortality globally, responsible for about 20.5 million deaths in 2021 alone [1]. They impose a substantial burden on healthcare systems, economies, and patients' lives and well‐being [2, 3].

Cardiac arrhythmias are particularly important, due to their rising prevalence, risk of complications, and marked effects on quality of life [3, 4]. Atrial fibrillation (AF) is the most common arrhythmia, affecting over 33 million individuals worldwide. While AF is frequently associated with underlying cardiovascular disease, it has become an important clinical focus, as untreated AF is associated with an increased risk of stroke, heart failure, and impaired quality of life [5, 6].

Treatment approaches include pharmacological therapy, direct current cardioversion, and cardiac ablation procedures, including catheter ablations. Catheter ablation is now an established intervention for various arrhythmias, including AF and ventricular tachycardia (VT).

The field continues to evolve, with novel energy sources such as pulsed‐field ablation (PFA) in 2019 [7, 8], introducing distinct anaesthetic considerations. As techniques develop, understanding the impact of different anaesthetic techniques becomes increasingly important.

These procedures require anaesthesia or sedation to ensure patient comfort and optimal conditions, with techniques including general anaesthesia (GA), deep sedation (DS), and conscious sedation (CS). However, the optimal approach remains an ongoing debate [9, 10].

Previous research has mainly evaluated specific anaesthetic techniques or selected patient populations. Despite important insights, no comprehensive synthesis of anaesthetic techniques across catheter ablation exists.

This scoping review systematically maps current evidence on anaesthetic methods employed in catheter ablations. It characterises the range of anaesthetic techniques, examines reported complications and recovery outcomes, identifies geographic variations, explores patient and operator perspectives, and describes outcome measures used to evaluate anaesthetic quality, thereby providing a foundation for future research.

## 2 Methods

This scoping review was conducted in accordance with the Preferred Reporting Items for Systematic Reviews and Meta‐Analysis, extensions for scoping reviews (PRISMA‐ScR) [11] and the previously published protocol [12].

### 2.1 Research Questions

1. Which anaesthetic techniques are currently used for catheter ablations?
2. What are the reported complications or adverse events associated with different anaesthetic techniques for catheter ablations?
3. How do the anaesthetic techniques facilitate postprocedural recovery?
4. Are there differences in patient satisfaction or operator satisfaction depending on the anaesthesia method used?
5. Are there variations in anaesthesia practices across geographic regions or healthcare settings?
6. What are the most reported outcome measures used to assess anaesthesia effectiveness and quality in these procedures?

### 2.2 Eligibility Criteria

The eligibility criteria were defined according to the population, concept and context (PCC) method:

#### 2.2.1 Population

Adult patients (age over 18) undergoing catheter ablation procedures.

#### 2.2.2 Concept

All kinds of anaesthesia and sedative techniques are used for catheter ablation.

#### 2.2.3 Context

Catheter ablation procedures are performed in hospitals, clinics, or similar healthcare facilities.

### 2.3 Search Strategy

The search strategy was developed in collaboration with an experienced information specialist (Appendix A, Tables A1, A2, A3). The final search string was refined and adapted for use on each database. We searched MEDLINE, EMBASE, Cochrane, Google Scholar (via ‘Publish or Perish’ [13]), and Scopus. The last two databases include grey literature. No search limits such as language, publication year, or study design were used. We included all primary studies where the primary outcome involved anaesthetic techniques. We excluded editorials and letters. Studies could include data from other electrophysiology (EP) procedures but were excluded if they did not present data from catheter ablations alone. Backwards and forwards snowballing was carried out on all included studies.

### 2.4 Selection of Sources of Evidence

All citations were uploaded to Covidence. The selection process consisted of two phases both carried out by two independent reviewers: title and abstract screening, followed by full‐text screening against the inclusion criteria (Appendix B). Any disagreements were resolved through discussion with the second reviewer.

### 2.5 Data Charting

Data charting was done by using a data extraction sheet in Excel (Table C1). The charting was performed by one reviewer, but to assure quality, data from 10% (11 articles) were checked by a random sample by another reviewer.

### 2.6 Assessment of Risk of Bias and Certainty of Evidence

We aimed to assess certainty of evidence using a modified GRADE (Grading of Recommendations Assessment, Development and Evaluation) approach, as applied in previous scoping reviews [14, 15, 16]. However, this was not deemed feasible during the review process.

### 2.7 Synthesis

This review sought to comprehensively map the existing evidence on the different anaesthetic techniques used for catheter ablations, guided by the key research questions described above. The analysis was conducted using a descriptive approach.

### 2.8 Artificial Intelligence Declaration

Artificial intelligence (OpenAI, ChatGPT 5) was used solely to assist with grammar checking throughout the manuscript, language refinement in the results section, and translation of non‐English and non‐Scandinavian articles. It was not used to generate scientific content, analyse or interpret data, or produce the results or conclusions presented in this manuscript. All suggestions were reviewed before incorporation into the manuscript. The authors take full responsibility for the accuracy and originality of the publication's content.

## 3 Results

We included 110 studies in this scoping review dating from 1991 to 2025. See Tables 1, 2, 3.

**TABLE 1:** Distribution of different study types included in this review.

Study type	Number of studies
Randomised controlled trial (RCT)	22 (20%)
Prospective observational	23 (21%)
Retrospective observational	28 (25%)
Prospective cohort	9 (8%)
Retrospective cohort	16 (15%)
Case–control	2 (2%)
Case report/case series	4 (4%)
Other	6 (5%)

**TABLE 2:** Number of studies regarding their published year.

Year	Number of studies
1990–1999	3 (3%)
2000–2009	7 (6%)
2010–2019	37 (34%)
2020–2025	63 (57%)

**TABLE 3:** Distribution of regions where the included studies originated from.

Region	Number of studies
Asia	27 (25%)
Europe	54 (49%)
Oceania	2 (2%)
North America	18 (16%)
Middle East	5 (4%)
South America	3 (3%)
Worldwide	1 (1%)

*Note:* Region reported is where the procedures took place. If not stated in article, country of 1st author was used.

### 3.1 Current Anaesthetic Techniques

Across the included studies, three techniques predominated: CS [17, 18, 19, 20, 21, 22, 23, 24, 25, 26, 27, 28, 29, 30, 31, 32, 33, 34, 35, 36, 37, 38, 39, 40, 41, 42, 43, 44, 45, 46, 47, 48, 49, 50, 51, 52, 53, 54, 55, 56, 57, 58, 59, 60, 61, 62], GA [17, 19, 20, 21, 23, 26, 35, 38, 39, 43, 44, 46, 49, 50, 52, 54, 55, 57, 58, 59, 61, 63, 64, 65, 66, 67, 68, 69, 70, 71, 72, 73, 74, 75, 76, 77, 78, 79, 80, 81, 82, 83, 84, 85, 86, 87, 88, 89] and DS [25, 31, 35, 38, 44, 61, 62, 69, 75, 80, 84, 87, 90, 91, 92, 93, 94, 95, 96, 97, 98, 99, 100, 101, 102, 103, 104, 105, 106, 107, 108, 109, 110, 111, 112, 113, 114, 115, 116, 117, 118, 119]. Lighter techniques such as mild/minimal sedation [66, 74, 77, 90, 116, 120] and local anaesthesia (LA) [83, 105] were rarely investigated. Only a single case study reported spinal anaesthesia [121]. See American Society of Anesthesiology (ASA)'s definitions in Table D1.

**FIGURE 1:**
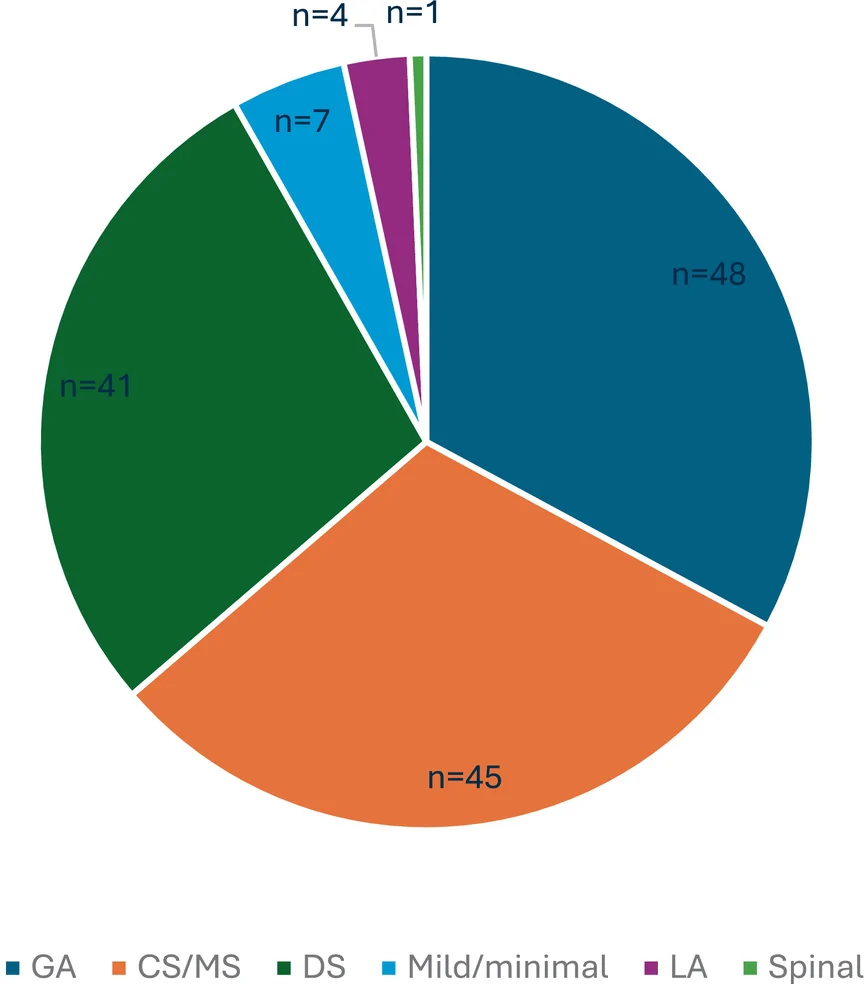
Distribution of anaesthetic techniques used for catheter ablations across the included studies. General Anaesthesia (GA), Deep Sedation (DS), Conscious Sedation (CS), Moderate Sedation (MS), Local Anaesthesia (LA).

**FIGURE 2:**
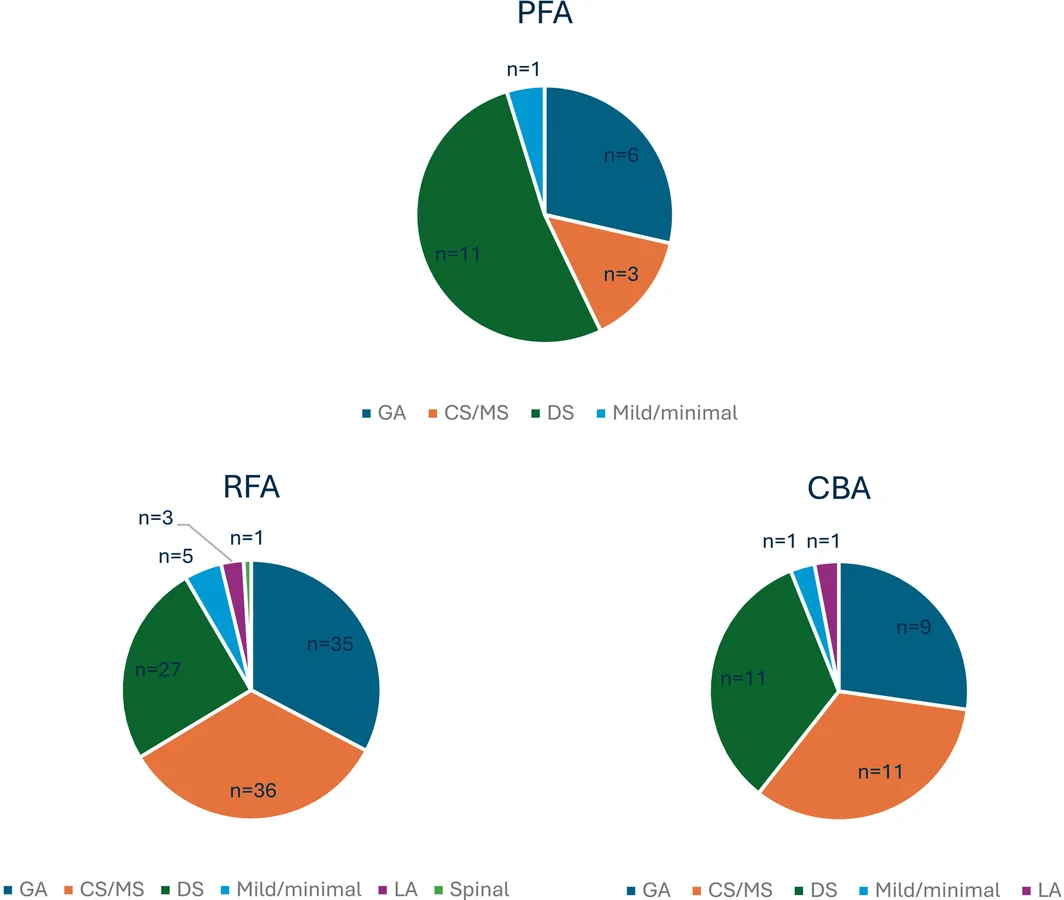
Distribution of different anaesthetic techniques used for catheter ablation, regarding ablation type. Radiofrequency ablation (RFA), cryoballoon ablation (CBA) and pulsed field ablation (PFA).

**FIGURE 3:**
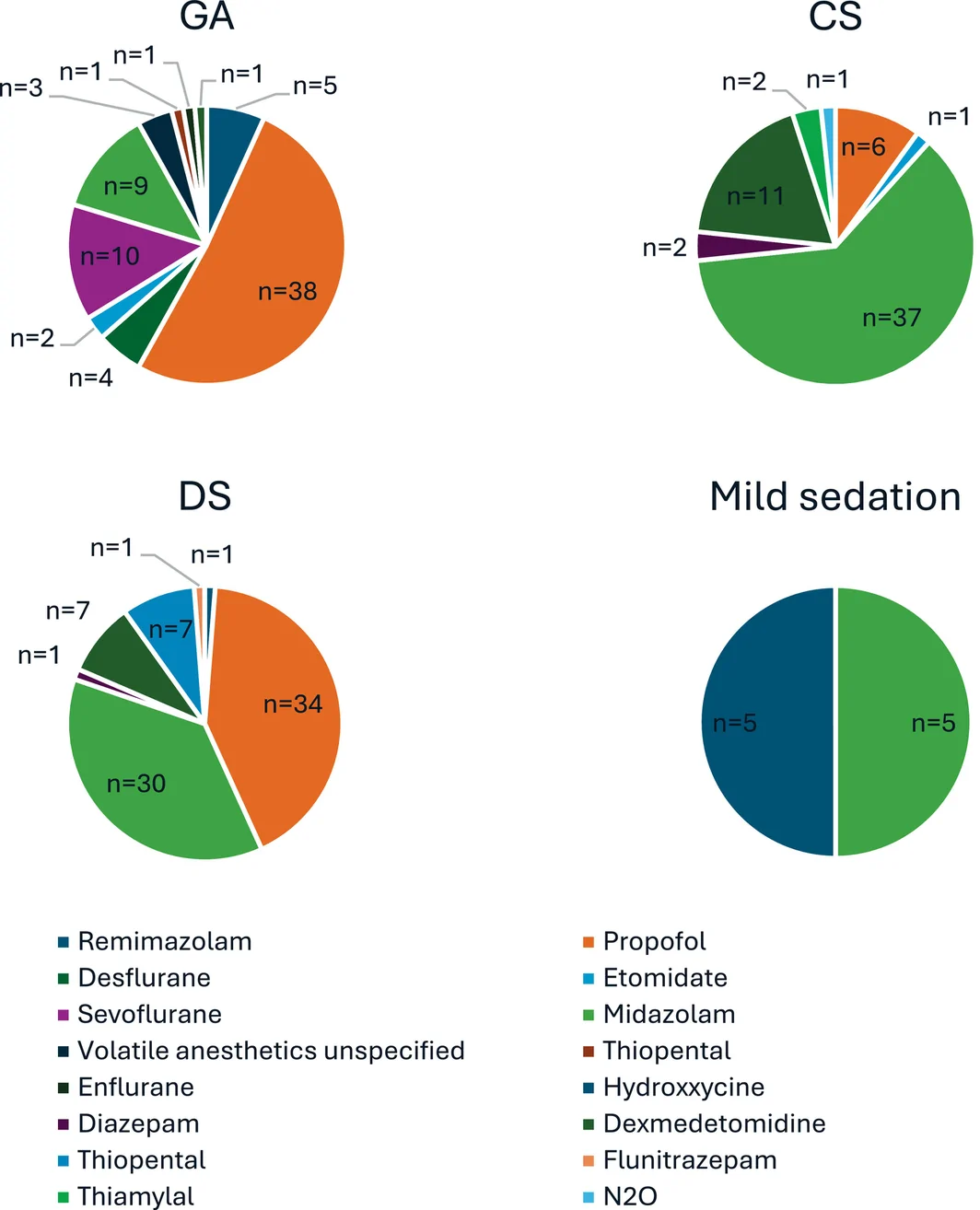
Distribution of sedatives in regard to anaesthesia technique. No distinction was made between drugs being used for induction or maintenance, or whether they were administered in combination.

The distribution of anaesthetic techniques is illustrated in Figure 1 (all studies), Figure 2 (by ablation type), and Figure 3 (sedatives by technique).

Definitions of sedation levels varied across studies. About a third of GA studies provided explicit sedation level definitions [17, 19, 20, 26, 35, 38, 44, 49, 52, 63, 64, 65, 66, 70, 72, 73, 76, 80, 84, 85], most commonly based on Bispectral Index (BIS) values 40–60. Despite variation in wording, definitions in the remaining studies regarding GA aligned with ASA's definition.

CS was more frequently used than MS (moderate sedation), with approximately half providing formal definitions [17, 18, 19, 22, 26, 29, 31, 35, 36, 38, 40, 41, 42, 44, 47, 51, 52, 53, 58, 59, 60]. Sedation level was usually assessed clinically, with patients described as having a drug‐induced depression of consciousness, while purposefully responding to verbal commands, with or without light tactile stimulation, and breathing spontaneously. One study deviated from the overall pattern, describing CS as patients being perfectly awake [41]. A range of scales were used, including Richmond Agitation‐Sedation Scale (RASS) [122] −2 to −3, Ramsay Sedation Scale (RSS) [123] 2–4 or under 3, Observer's Assessment of Alertness/Sedation (OOA/S) [124] 3–4, Sedation level (SED) 8 [42] and BIS 71–90.

About half of DS studies defined sedation depth, though methods varied [31, 35, 38, 44, 69, 80, 91, 92, 94, 95, 96, 97, 99, 100, 103, 106, 108, 112, 113, 114, 115, 116, 117, 118]. Some studies used ASA's definition [125], whereas more than half used standardised scales such as RSS from 3 to 6, Modified Observer's Assessment of Alertness and Sedation (MOOA/S) [126] 3, RASS −5 to −4, BIS values 40–80 and Patient State Index (PSI) < 70.

Minimal/mild Sedation was defined following ASA's definition [120] or RSS scores 3–4 [116, 120], while LA was only defined in one study as no loss of consciousness but reduced discomfort [105].

Overall, heterogeneity was observed in how sedation levels were defined and assessed, with inconsistent terminology and variable use of formal scoring systems.

### 3.2 Complications and Adverse Events

No serious sedation‐related complications or adverse events were reported, except for one case series focusing on difficult ventilation because of potential laryngospams [86].

Overall, sedation‐related complications were infrequently observed, and none were reported in studies using minimal/mild sedation or LA [46, 83, 105].

Hypotension was the most frequently observed across GA, CS, and DS, followed by hypoxia/desaturation, particularly in DS and CS. Other adverse events were only occasionally reported and included vomiting or hallucinations during the procedure in a study using CS [41], as well as postprocedural pneumonia in elderly patients in a study using DS [104]. One study reported conversion from DS to GA in a great number of patients mainly due to persistent hypotension [99].

Comparative analysis suggested fewer adverse events such as cough, hypoxemia and hypotension in GA versus DS [69, 80] and in CS versus DS [31], while similar rates were observed between GA and CS [17, 20, 21, 26, 39, 43, 52, 54, 57, 58]. An exception was a higher occurrence of unstable VTs in ablation for scar‐related VT under GA [39].

Taken together, sedation‐related complications and adverse events were uncommon, with hypotension and hypoxia being the most consistently reported.

### 3.3 Postprocedural Recovery

About a quarter of the studies investigated postprocedural recovery, generally reporting short recovery times across anaesthetic techniques [18, 22, 24, 25, 31, 41, 42, 43, 63, 64, 65, 70, 72, 74, 82, 83, 84, 91, 92, 93, 98, 99, 100, 104, 110, 119, 127, 128]. Assessment methods varied, with some measuring time to satisfactory scores on formal scales, like RSS, Steward Recovery score [129] or (modified) Aldrete scale [130], while others used time‐based measures like ‘time to’ extubation, eye‐opening, or laboratory exit or documented delayed emergence. One study assessed recovery quality using the Korean version of the Quality of Recovery‐15 (QoR‐K) score [131] and found similarly high recovery quality between remimazolam/remifentanil and propofol/remifentanil [63].

Comparative findings suggested differences between techniques. CS was associated with shorter emergence times than DS [31], while DS demonstrated shorter completion‐to‐exit time than GA [84]. Overall, recovery profiles for CS and GA appeared comparable, with no evidence of prolonged or complicated recovery.

Differences were also observed between sedative techniques. Propofol was generally associated with fast emergence and recovery, though remimazolam showed shorter ‘time to eye‐opening’ and ‘time to extubation’ [63]. In addition, patient‐controlled sedation with remifentanil was linked with shorter recovery time than propofol [127].

PONV was reported in a small proportion of patients across studies [18, 22, 31, 33, 41, 53, 56, 63, 64, 65, 69, 91, 92, 93, 106, 127, 132], though fentanyl/midazolam caused more nausea than propofol [31].

In general, recovery outcomes were favourable across anaesthetic techniques, with only minor differences observed between techniques.

### 3.4 Patient and Operator Satisfaction

Evidence on operator satisfaction was limited, with only 12 studies addressing this outcome [18, 22, 31, 47, 56, 74, 76, 80, 91, 92, 127, 132]. Most studies used numeric scales such as ‘Likert’ [133] or similar instruments. Despite this heterogeneity in assessment methods, operator satisfaction was generally high.

Comparative data were limited. Only two studies compared different techniques, with one reporting higher operator satisfaction with GA over DS [80], and another reporting higher operator satisfaction with CS compared to Unconscious Sedation (US) [31].

Some studies compared pharmacological agents, finding higher operator satisfaction with dexmedetomidine than midazolam for CS [18], and for deep over moderate neuromuscular blockade under GA [76].

Patient satisfaction was assessed in 21 studies [18, 19, 22, 30, 31, 41, 42, 47, 58, 60, 66, 74, 80, 91, 92, 93, 97, 98, 108, 115, 127]. Assessment tools predominantly included numerical scales or questionnaires, consistently demonstrating high satisfaction levels, low pain scores, and minimal anxiety or discomfort across anaesthetic techniques.

However, the available evidence suggested some variation between techniques and pharmacological agents. LA showed lower patient satisfaction than DS and CS for atrioventricular nodal re‐entry tachycardia (AVNRT)/atrioventricular re‐entry tachycardia/atrial tachycardia ablation [30, 98], while higher pain scores were reported with mild conscious sedation (mCS) and CS when compared to GA [57, 66], and with CS compared to DS [31]. Dexmedetomidine had a higher patient satisfaction than midazolam [18], but when dexmedetomidine was compared to propofol, it was associated with higher pain scores [97]. These findings were reflected in patient preferences within the same studies, with greater willingness to undergo the same sedation type with propofol over dexmedetomidine, and GA being more likely to be recommended over CS.

Findings related to anxiety and discomfort showed inconsistencies. Nalbuphine was linked to higher levels of anxiety and discomfort than N_2_O for CS [41]. When comparing mild sedation to GA, anxiety ratings varied by assessment method, with higher anxiety scores reported for mild sedation on a numerical scale, whilst GA was ‘worse than expected’ on a Likert scale [66]. Patients also reported more postprocedural throat pain after GA [66].

Across studies, both operator and patient satisfaction were generally favourable, although differences were observed depending on anaesthetic technique and pharmacological strategy.

### 3.5 Geographic and Healthcare Variations

Just over half of the studies reported procedural settings, almost all describing ablations being performed in an EP or catheter lab, thereby limiting comparison across different healthcare settings.

Regional variations in anaesthetic practice were evident. North American studies predominantly used GA, with fewer examining DS or CS, and were the only region reporting use of spinal anaesthesia [121].

In Asia, GA and CS were most frequently investigated, with moderate representation of DS and little focus on minimal sedation and LA.

European practice patterns deviated notably, with DS being the most frequently investigated technique, followed by CS and GA, while lighter sedation techniques remained similarly underexplored.

Evidence from South America and the Middle East was limited but suggested a more even distribution across sedation techniques. South American studies investigated GA, DS, and CS [42, 69, 85], while Middle Eastern studies examined CS, DS, and no sedation [25, 35, 88, 98, 119].

In Oceania, studies focused on GA and CS [52, 89].

Sedative preference also differed across regions. Europe and North American most frequently reported midazolam, followed by propofol, whereas Asia predominantly used propofol, followed by midazolam and dexmedetomidine.

The novel benzodiazepine remimazolam was explored primarily in Asian studies [63, 64, 65, 84], and only one European study [80].

In contrast, analgesic preferences were more uniform. Fentanyl was the most used analgesic in all regions followed by remifentanil, except South America where remifentanil predominated. Asia was the only region investigating pentazocine [20, 32, 84, 106, 116, 120].

Overall, regional variation was evident in anaesthetic techniques and sedative preferences while analgesic use appeared more consistent.

### 3.6 Reported Outcome Measures

Complications and adverse events, along with intraprocedural respiratory and haemodynamic stability, were commonly used as ways of assessing the anaesthesia quality.

**FIGURE 4:**
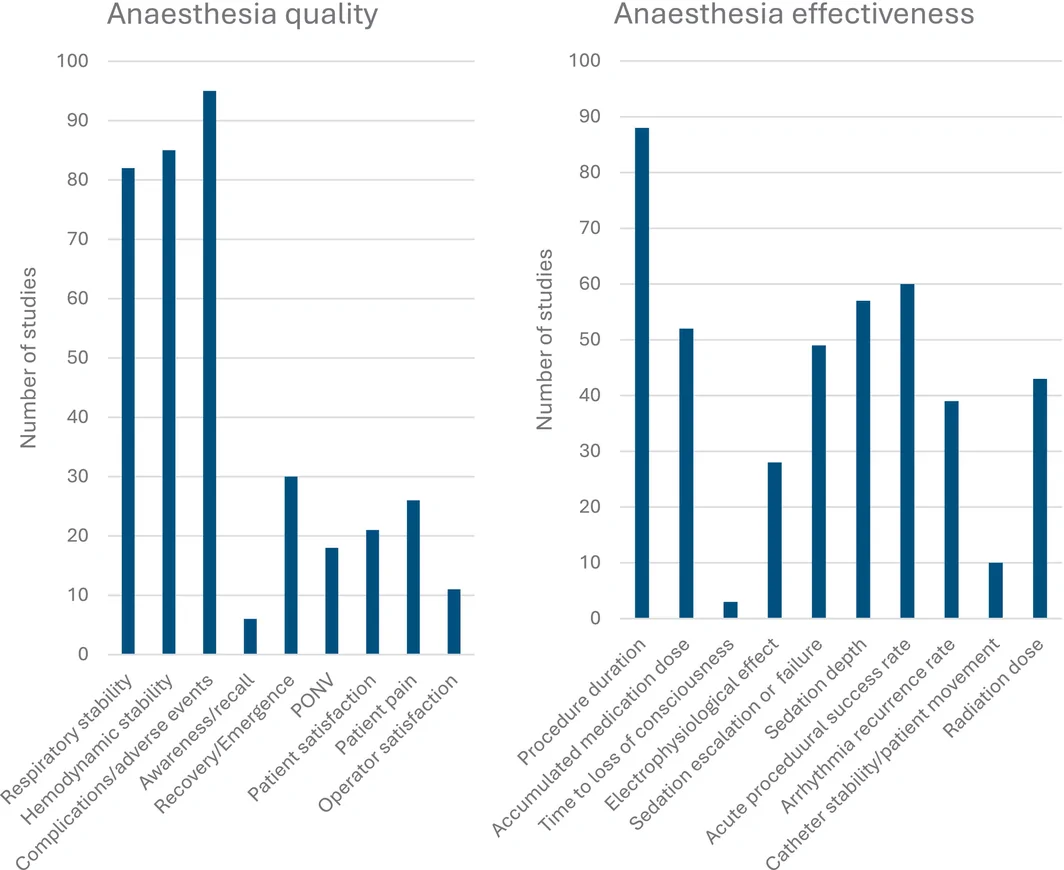
Outcome measures used to assess anaesthesia quality and effectiveness. Anaesthesia quality refers to the safety, comfort, and satisfaction associated with anaesthesia, including minimisation of adverse events and optimisation of patient and proceduralist experience. Anaesthesia effectiveness refers to how well anaesthesia achieves its physiological and procedural goals (adequate sedation, timely induction, optimal working conditions, etc.)

Effectiveness was most often assessed using procedure duration, though accumulated sedative/analgesic dose, acute procedural success rate, sedation depth, and sedation failure or escalation were also frequently employed. All reported outcome measures are shown in Figure 4.

## 4 Discussion

This scoping review identified substantial heterogeneity in anaesthetic practices, terminology, and outcome assessment across catheter ablation procedures. Most studies focused on GA, DS, and CS, with lighter sedation techniques rarely investigated. Reporting of complications, recovery, and satisfaction was inconsistent, limiting comparability between studies.

When examining AF ablations, the distribution of GA, CS, and DS was similar across studies. This contrasts with global trends reported by Garcia et al., who found increasing use of GA over time [44]. PFA is a newer and more painful method typically requiring deeper sedation. It was almost exclusively performed under DS, which may explain the relatively high representation of DS in our findings.

Only 16 studies examined PFA [59, 60, 61, 66, 75, 80, 86, 87, 91, 92, 94, 102, 108, 111, 113, 115], reflecting its recent introduction. Most PFA studies used DS or GA, while lighter sedation techniques remain insufficiently investigated. One study found mCS inadequate for pain control despite positive patient feedback [66], whereas two studies reported the feasibility of CS [59, 60].

Most included studies focused on AF, leaving limited evidence for other arrhythmias including VT, Wolf‐Parkinson‐White, AVNRT and atrial flutter [24, 25, 30, 34, 36, 37, 38, 39, 41, 42, 47, 48, 64, 65, 67, 68, 73, 78, 81, 83, 88, 89, 90, 93, 98, 100, 101, 107, 109, 112, 113, 117, 121, 127, 128]. This evidence gap and imbalance limit the generalizability of these conclusions to non‐AF ablations due to differences in duration, complexity, and therefore anaesthetic requirements.

The findings regarding complications, recovery, and satisfaction should be interpreted cautiously. Although serious sedation‐related complications were rarely reported, this cannot be interpreted as evidence of overall safety across anaesthetic techniques. Reporting was inconsistent, complications were often not predefined outcomes, and several studies only briefly described adverse events. Hypotension and respiratory events such as hypoxia or desaturation were the most consistently reported complications, particularly in DS and CS studies, whereas other outcomes such as nausea/vomiting, pneumonia, difficult ventilation, or conversion to GA were only sporadically addressed.

Recovery outcomes were similarly heterogeneous. Studies used different outcome measures including time to extubation, eye opening, laboratory exit, discharge readiness, and formal recovery scales, making comparison difficult. Consequently, the available evidence does not allow conclusions regarding whether one anaesthetic technique meaningfully improves recovery or procedural flow.

Patient and operator satisfaction were generally reported as high across techniques, but assessment methods varied considerably, and validated instruments were rarely used. These findings therefore primarily demonstrate that satisfaction outcomes are considered clinically relevant, rather than establishing superiority of specific anaesthetic techniques.

GA was the most investigated technique, followed by CS and then DS. Gaitan et al. [134] conducted a worldwide survey among cardiologists and mapped anaesthesia preferences across a range of EP procedures. They found cardiologists favouring sedation/MAC (monitored anaesthesia care) over GA for almost all EP procedures, except AF ablations and epicardial VT ablations. The large proportion of AF ablation studies in this review likely explains why GA accounted for a relatively high share of the reported techniques.

Few studies directly compared anaesthetic requirements across ablation modalities [27, 28, 48, 51, 62, 74, 102, 109, 111, 114]. Available evidence may suggest that RFA may require greater analgesic and sedative support than CBA [48, 114], while findings for PFA remain inconsistent [111]. As PFA is considered more painful, but often shorter in duration, further comparative studies are needed.

Approximately half of the included studies defined sedation levels, with considerable variation and overlaps in definitions. Some studies used the term mCS, which is not an official ASA category [66, 74, 77]. Only one study using this term provided a definition, contributing to inconsistency and obscuring sedation depth, thereby limiting comparability across studies.

Operator‐relevant factors, like anaesthesia's electrophysiological effect [20, 30, 34, 36, 39, 40, 42, 43, 47, 49, 54, 67, 68, 73, 81, 83, 84, 88, 89, 96, 98, 101, 105, 112, 116, 121, 128, 135] and catheter stability/patient movement were infrequently addressed [26, 31, 32, 57, 69, 75, 76, 84, 110, 118]. Only one study reported interruptions due to movement as a complication [118].

Only two studies examined satisfaction among anaesthesia personnel [47, 80].

Time to loss of consciousness [63, 64, 91] and patient awareness/recall [60, 63, 93, 108, 115, 128] was minimally assessed, despite playing a significant role in patient experience. Other duration parameters including total anaesthesia time [20, 31, 62, 64, 65, 70, 71, 72, 76, 82, 91, 92, 109, 127, 136], total lab time [20, 23, 26, 75, 91, 94] and non‐procedure time [23, 55, 66, 84, 94] were reported in a small number of studies. Comparing techniques on these parameters could impact OR flow and potentially increase patient turnover.

Another unexplored area was the cost‐effectiveness of the different anaesthetic techniques [21, 55, 71, 83]. This represents a meaningful direction for future research, as optimising anaesthetic techniques could reduce healthcare costs.

### 4.1 Strengths and Limitations

This scoping review has several strengths and follows a published protocol and the PRISMA‐ScR guidelines, using a broad and systematic search strategy developed with an experienced health science information specialist. The search covered multiple databases and was supplemented by backwards and forwards snowballing on all the included articles. No studies were excluded because of language or publication year, eliminating language and publication bias.

However, there were some limitations. The screening process was carried out by two independent reviewers, but data extraction was performed by only one reviewer and checked by another, which could increase the risk of errors. The broad inclusion criteria provide extensive mapping of existing literature, but in‐depth analysis is lacking, making it difficult to draw specific and actionable conclusions. A methodological limitation was that many studies lacked comparison groups, hindering comparative evaluation of anaesthetic techniques.

Articles not written in English or Scandinavian languages were translated using AI‐based translation tools and some nuance or detail may have been lost in translation. Consistent with the scoping review methodology, no risk of bias assessment was conducted.

In addition, interpretation of complications, recovery, and satisfaction outcomes was limited by inconsistent definitions, heterogeneous reporting methods, and lack of standardised outcome measures. As no formal risk‐of‐bias assessment was performed, the trustworthiness of comparative findings remains uncertain, and these findings should therefore be interpreted with caution.

Despite these limitations, this review offers broad and valuable insight into the existing evidence on applied anaesthesia techniques in catheter ablations, highlighting key evidence gaps.

## 5 Conclusion

This scoping review mapped current anaesthetic practices in catheter ablations and identified substantial variation in techniques, terminology, and outcome assessment. GA, DS, and CS were the most frequently reported techniques, while lighter sedation techniques were rarely investigated.

Evidence gaps remain regarding comparative safety, recovery, patient experience, and workflow‐related outcomes. Standardised definitions and outcome reporting are needed to improve comparability and guide future research.

## Author Contributions

All authors contributed to the development of the manuscript. M.B.F. and A.F.P. screened all studies. Data extraction was performed by M.B.F. and checked by A.F.P. M.B.F. drafted the manuscript and all authors critically revised and approved the final version.

## Funding

The authors have nothing to report.

## Conflicts of Interest

The authors declare no conflicts of interest.

## Data Availability

The data that support the findings of this study are available from the corresponding author upon reasonable request.

## Appendix A

### Search Strings

**TABLE A1:** MEDLINE the 21st of January 2026.

#	Query	Results
#1	(ablat* adj3 techniq*).mp.	9667
#2	ablation techniques/ or cryosurgery/ or radiofrequency ablation/ or catheter ablation/ or maze procedure/	62,241
#3	((Catheter* adj2 ablat*) or (Radiofrequenc* adj2 ablat*) or Cryoablat* or (pulse* adj2 field* adj2 ablat*) or (Pulmon* adj2 Vein* adj2 isolat*) or RFA or PFA).mp.	72,846
#4	(An?esth* or Anaesth* or Anesth*).mp.	540,337
#5	sedat*.mp.	99,078
#6	exp anesthesia/ or conscious sedation/ or deep sedation/	223,660
#7	#4 OR #5 OR 6	624,453
#8	exp Arrhythmias, Cardiac/	253,235
#9	(arrhythmi* or (Atria* adj fib*) or (Atria* adj flutter*) or (Atrioventricular adj3 tachycardi*) or Wolff‐Parkinson‐White syndrome or (Ventric* adj2 tachycardi*) or Ventric* ectopic* beat* or Premature ventric* contraction* or Sinus node reentr* tachycardi*).mp.	301,064
#10	#8 OR #9	361,482
#11	#1 OR #2 OR #3	88,846
#12	#7 AND #10 AND #11	855

### Scopus the 23th of January 2026

((ablat* W/2 techniq*) OR (Catheter* W/2 ablat*) OR (Radiofrequenc* W/2 ablat*) OR Cryoablat* OR (pulse* W/2 field* W/2 ablat*) OR (Pulmon* W/2 Vein* W/2 isolat*) OR RFA OR PFA) AND (Anaesth* OR Anesth* OR Sedat*) AND (arrhythmi* OR (Atria* W/1 fib*) OR (Atria* W/1 flutter*) OR (Atrioventricular W/3 tachycardi*) OR “Wolff‐Parkinson‐White syndrome” OR (Ventric* W/2 tachycardi*) OR “Ventric* ectopic* beat*” OR “Premature ventric* contraction*” OR “Sinus node reentr* tachycardi*”).

11,113 hits.

### Google Scholar Via Publish or Perish the 21st of January

(“catheter ablation” OR “radiofrequency ablation” OR cryoablation OR “pulsed field ablation” OR RFA OR PFA) AND (anesthesia OR anaesthesia OR sedation) AND (arrhythmia OR “atrial fibrillation” OR “ventricular tachycardia”).

1000 hits.

**TABLE A2:** Cochrane the 21st of January 2026.

ID	Search	Hits
#1	MeSH descriptor: [Anesthesia] explode all trees	25,557
#2	MeSH descriptor: [Arrhythmias, Cardiac] explode all trees	14,151
#3	MeSH descriptor: [Ablation Techniques] explode all trees	8541
#4	((Catheter*:ti,ab,kw NEAR/2 ablat*:ti,ab,kw) OR (Radiofrequenc*:ti,ab,kw NEAR/2 ablat*:ti,ab,kw) OR Cryoablat*:ti,ab,kw OR (pulse*:ti,ab,kw NEAR/2 field*:ti,ab,kw NEAR/2 ablat*:ti,ab,kw) OR (Pulmon*:ti,ab,kw NEAR/2 Vein*:ti,ab,kw NEAR/2 isolat*:ti,ab,kw) OR RFA:ti,ab,kw OR PFA:ti,ab,kw)	8270
#5	(ablat*:ti,ab,kw NEAR/3 techniq*:ti,ab,kw)	730
#6	(An?esth*:ti,ab,kw OR Anaesth*:ti,ab,kw OR Anesth*:ti,ab,kw)	135,676
#7	sedat*:ti,ab,kw	35,817
#8	(arrythmi*:ti,ab,kw OR (Atria*:ti,ab,kw NEAR/2 fib*:ti,ab,kw) OR (Atria*:ti,ab,kw NEAR/2 flutter*:ti,ab,kw) OR (Atrioventricular:ti,ab,kw NEAR/3 tachycardi*:ti,ab,kw) OR “Wolff‐Parkinson‐White syndrome”:ti,ab,kw OR (Ventric*:ti,ab,kw NEAR/2 tachycardi*:ti,ab,kw) OR (Ventric* NEAR/2 ectopic* NEAR/2 beat*):ti,ab,kw OR (“Premature” NEAR/2 ventric* NEAR/2 contraction*):ti,ab,kw OR (“Sinus node” NEAR/2 reentr* NEAR/2 tachycardi*):ti,ab,kw)	21,254
#9	#1 OR #6 OR #7	156,155
#10	#2 OR #8	25,722
#11	#3 OR #4 OR #5	14,157
#12	#9 AND #10 AND #11	174

**TABLE A3:** EMBASE the 21st of January 2026.

ID	Query	Results
#1	(ablat* adj3 techniq*).mp.	8995
#2	((Catheter* adj2 ablat*) or (Radiofrequenc* adj2 ablat*) or Cryoablat* or (pulse* adj2 field* adj2 ablat*) or (Pulmon* adj2 Vein* adj2 isolat*) or RFA or PFA).mp.	135,690
#3	(An?esth* or Anaesth* or Anesth*).mp.	812,209
#4	sedat*.mp.	183,423
#5	(arrythmi* or (Atria* adj fib*) or (Atria* adj flutter*) or (Atrioventricular adj3 tachycardi*) or Wolff‐Parkinson‐White syndrome or (Ventric* adj2 tachycardi*) or Ventric* ectopic* beat* or Premature ventric* contraction* or Sinus node reentr* tachycardi*).mp.	369,024
#6	exp radiofrequency ablation/ or exp. catheter ablation/ or exp. thermal ablation/ or exp. ablation catheter/ or exp. radiofrequency catheter ablation/	105,323
#7	exp continuous epidural anesthesia/ or exp. “delayed emergence from anesthesia”/ or exp. opioid‐free anesthesia/ or exp. anesthesia induction/ or exp. anesthesia complication/ or exp. anesthesia information system/ or exp. anesthesia/ or exp. anesthesia nursing/ or exp. regional anesthesia/ or exp. topical anesthesia/ or exp. inhalation anesthesia/ or exp. anesthesia mechanism/ or exp. low flow anesthesia/ or exp. balanced anesthesia/ or exp. intravenous regional anesthesia/ or exp. anesthesia level/ or exp. general anesthesia/ or exp. spinal anesthesia/ or exp. cardiac anesthesia/ or exp. epidural anesthesia/ or exp. intravenous anesthesia/ or exp. local anesthesia/ or exp. “anesthesia (sensory dysfunction)”/	508,489
#8	exp heart ventricle arrhythmia/ or exp. heart arrhythmia/ or exp. atrial fibrillation/	790,897
#9	1 or 2 or 6	142,660
#10	3 or 4 or 7	988,201
#11	5 or 8	807,146
#12	9 and 10 and 11	4610

## Appendix B

The PRISMA flowchart illustrates the stages of a systematic literature search, showing the number of studies identified, screened, assessed for eligibility and included in the final review, as well as the reasons for exclusion at various stages.

## Appendix C

**TABLE C1:** The used data extraction form.

Study characteristics	1st Author Year Title Country Setting Study type Comparison groups
Population	Age Gender ASA Sample size Arrhythmia(s)
Procedure	Ablation type Repeat ablations included Duration
Anaesthesia	Type Definition of sedation level Intubation Administered by Medications used Bolus/infusion Accumulated dose Other medication used for anaesthesia Sedation/anaesthesia related complications Other complications Need for change/escalation of sedation Emergence/recovery time Nausea/vomiting Patient and operatory satisfaction Outcome measures used to assess anaesthesia quality
Satisfaction	Patient Operator Assessment method
Outcome measures used to assess anaesthesia quality and effectiveness	—
Other/comment	—

## Appendix D

**TABLE D1:** Statement on continuum of depth of sedation: definition of general anesthesia and levels of sedation/analgesia [125].

	Minimal sedation anxiolysis	Moderate sedation/analgesia (“Conscious Sedation”)	Deep sedation/analgesia	General anesthesia
Responsiveness	Normal response to verbal stimulation	Purposeful** response to verbal or tactile stimulation	Purposeful** response following repeated or painful stimulation	Unarousable even with painful stimulus
Airway	Unaffected	No intervention required	Intervention may be required	Intervention often required
Spontaneous Ventilation	Unaffected	Adequate	May be inadequate	Frequently inadequate
Cardiovascular Function	Unaffected	Usually maintained	Usually maintained	May be impaired

*Note:* Minimal sedation (Anxiolysis) is a drug‐induced state during which patients respond normally to verbal commands. Although cognitive function and physical coordination may be impaired, airway reflexes, and ventilatory and cardiovascular functions are unaffected. This is typically accomplished by a single oral dose of a sedative or an analgesic administered before the procedure. Moderate Sedation/Analgesia (“Conscious Sedation”) is a drug‐induced depression of consciousness during which patients respond purposefully to verbal commands, either alone or accompanied by light tactile stimulation. No interventions are required to maintain a patent airway, and spontaneous ventilation is adequate. Cardiovascular function is usually maintained. This is typically accomplished by titration of IV sedatives and/or analgesics during the procedure. Deep Sedation/Analgesia is a drug‐induced depression of consciousness during which patients cannot be easily aroused but respond purposefully following repeated or painful stimulation. The ability to independently maintain ventilatory function may be impaired. Patients may require assistance in maintaining a patent airway, and spontaneous ventilation may be inadequate. Cardiovascular function is usually maintained. This is typically accomplished by titration of IV sedatives and/or analgesics and/or anesthetics during the procedure. General Anesthesia is a drug‐induced loss of consciousness during which patients are not arousable, even by painful stimulation. The ability to independently maintain ventilatory function is often impaired. Patients often require assistance in maintaining a patent airway, and positive pressure ventilation may be required because of depressed spontaneous ventilation or drug‐induced depression of neuromuscular function. Cardiovascular function may be impaired. ** Reflex withdrawal from a painful stimulus is NOT considered a purposeful response.
